# Development and external validation of a prognostic nomogram for acute decompensation of chronic hepatitis B cirrhosis

**DOI:** 10.1186/s12876-018-0911-y

**Published:** 2018-12-03

**Authors:** Fangyuan Gao, Xiaoshu Li, Gang Wan, Yuxin Li, Qun Zhang, Yao Liu, Huimin Liu, Hai Li, Xianbo Wang

**Affiliations:** 1 Center of Integrative Medicine, Beijing Ditan Hospital, Capital Medical University, Beijing, China; 2 Statistics Room, Beijing Ditan Hospital, Capital Medical University, Beijing, China; 3 Department of Gastroenterology, Ren Ji Hospital, School of Medicine, Shanghai Jiao Tong University, Shanghai, China

**Keywords:** Chronic liver disease, Chronic hepatitis B, Nomogram, Prognosis

## Abstract

**Background:**

Acute decompensation (AD) has been shown to be associated with a high mortality rate for cirrhosis patients. This study aimed to develop a prognostic nomogram to evaluating the individual prognosis for AD of cirrhosis in chronic hepatitis B (CHB).

**Methods:**

The nomogram was developed using data from a retrospective study on 509 patients hospitalized for AD of CHB cirrhosis from October 2008 to February 2014 at the Beijing Ditan Hospital, Capital Medical University. The predictive accuracy, discriminative ability, and clinical net benefit were evaluated by concordance index (C-index), calibration curves, and decision curve analysis (DCA). The results were validated on 620 patients consecutively enrolled from January 2005 to December 2010 at the Renji Hospital, Shanghai Jiao Tong University,.

**Results:**

On multivariate analysis of the derivation cohort, independent factors included in the nomogram were age, previous decompensation, bacterial infection, hepatic encephalopathy, and total bilirubin. The calibration curve for the probability of survival showed good agreement between the nomogram and actual observation. The nomogram had a C-index of 0.897, which was statistically higher than the C-index values of CTP (0.793), MELD (0.821), SOFA (0.868), or the Chronic Liver Failure Consortium AD (CLIF-C AD) (0.716) scores (*p* <  0.001 for all). Using DCA, the nomogram also demonstrated superior net benefits over other score models. The results were confirmed in the validation cohort.

**Conclusions:**

The proposed nomogram enables more-accurate individualized prediction of survival than MELD, CTP, SOFA, or CLIF-C AD scores for AD of CHB cirrhosis patients.

## Background

Hepatitis B virus (HBV) infection is a critical global health threat, particularly in Asia. In the natural history of chronic HBV infection, partial patients remain asymptomatic until an episode of acute decompensation (AD), which is featured by the rapid development of one or more major complications of liver disease. AD is the common cause of hospital admission and is associated with a high mortality rate for cirrhosis patients. AD of cirrhosis occurs in as many as 15% of cirrhosis patients each year, and the frequency and severity will increase with the progressively deterioration of liver reserve function. The course of illness during a patient’s early hospital phase has been related to eventual prognosis. Therefore, early recognition and aggressive treatment are important to improving survival [1–4].

Recently, the CANONIC research team built the Chronic Liver Failure Consortium AD (CLIF-C AD), which was demonstrated to be superior to the Model for End-stage Liver Disease (MELD), MELD-Na, and Child-Turcotte-Pugh (CTP) assessments in predicting mortality due to AD [5]. However, the current score models were built primarily base on populations with alcoholism issues and/or hepatitis C virus infections [5]. To date, no reports of a model have been found to assess the prognosis of AD of chronic hepatitis B (CHB) cirrhosis. CHB cirrhosis is deemed as the leading cause of AD in the Asia-Pacific region [6]. Therefore, it is necessary to create a risk model that can predict the prognosis for AD of CHB cirrhosis.

The nomogram is a graphical calculating and two-dimensional diagram, which could offer more precise and individualized prediction relative to the traditional models for multiple illness [6–9]. The specific objective of the study was to determine the risk factors for short-term death of AD with CHB cirrhosis, develop a prognostic nomogram to estimate the individual prognosis, and promote the implementation of preventive measures.

## Patients and methods

### Derivation cohort

In total, 1781 patients hospitalized at Beijing Ditan Hospital (Beijing, China), which is affiliated with the Capital Medical University, between October 2008 and February 2014 with CHB cirrhosis were screened, and 509 patients with AD of CHB cirrhosis were enrolled for model derivation. All patients were limited to age ≥ 18 years in this study, and patients with the following diseases would be excluded: (1) infected with hepatitis virus (including A, C, D, and E), human immunodeficiency virus, or other viruses; (2) autoimmune hepatitis, drug-induced liver injury, alcohol-induced liver disease, Wilson’s disease, liver cancer, or hemolytic diseases; (3) other fatal disease, or gestation; or (4) liver operation or liver transplantation. The Ethics Committee from Beijing Ditan Hospital has approved this study protocol.

### Validation cohort

2610 patients hospitalized with CHB cirrhosis at Renji Hospital, Shanghai Jiao Tong University, from January 2005 to December 2010 were screened, and 620 patients with AD of CHB cirrhosis were enrolled for external validation of the model. Only patients meeting the inclusion and exclusion standard and having sufficient data were registered. The research was supported by the Ethics Committee from Renji Hospital.

Cirrhosis was confirmed by previous hepatic pathology or clinical signs and results of laboratory detection, radiologic and endoscopic presentation.

AD of cirrhosis was defined as the rapid development of one or more major complications (ie, large ascites, hepatic encephalopathy (HE), gastrointestinal hemorrhage and bacterial infection) of liver disease [10–14]. Cirrhosis patients with grade 2 to 3 ascites within 2 weeks could be diagnosed with the acute development of large ascites [10]. Acute HE was characterized by the development of the acute confusional status in patients with previously normal conscious state and no evidence of the neurological deficit [11]. Cirrhosis patients with occurrence of upper or lower gastrointestinal bleeding could be diagnosed with acute gastrointestinal hemorrhage [12]. Bacterial infection was defined to include spontaneous bacterial peritonitis (SBP), pneumonia, cellulitis, urinary system infection and spontaneous bacteremia [13].

Definitions of organ failures were as follows [15]: the definition of liver failure was the total bilirubin (TBil) ≥ 12.0 mg/dL; the definition of kidney failure was the serum creatinine (Cr) ≥ 2.0 mg/dL or the use of renal replacement therapy; cerebral failure was defined by grade III-IV HE; the definition of coagulation failure was an international normalized ratio (INR) ≥ 2.5; the definition of circulatory failure was the use of vasoconstrictors; the definition of respiratory failure was PaO_2_/FiO_2_ ≤ 200 or SpO_2_/FiO_2_ ≤ 214.

The MELD, CTP, SOFA and CLIF-C AD scores were calculated according to previously published criteria [5, 16–18]. All definitions and prognostic scores were applied at baseline.

### Treatment

Standard medical therapies were used for all patients after diagnosis, such as bed rest, liver-protecting therapy, antiviral treatment, nutritional support, vitamins supplements, plasma and albumin transfusion, maintenance treatment of water-electrolyte and acid-base balance, control and prevention of complications.

### Clinical predictors

Possible variables associated with short-term survival of AD of CHB cirrhosis were collected, including age; sex; mean arterial pressure; decompensation history; occurrences of organ failures or clinic complications; biochemical parameters, including alanine aminotransferase (ALT), aspartate aminotransferase (AST), γ-glutamyl transpeptidase (GGT), albumin (ALB), TBil, Cr, serum Na, INR, white blood cell (WBC) count, absolute neutrophil count (NC), absolute platelet count (PLT), and HBV deoxyribonucleic acid (DNA) levels. Baseline data were obtained at the first diagnosis of AD of CHB cirrhosis. The 7-, 14- and 28-day survival rates were gained by searching the medical records.

### Statistical analysis

Statistical analysise were conducted using SPSS version 20.0 (IBM, Armonk, NY, USA). Patient characteristics were compared by the Fisher’s exact or χ ^2^ tests (categorical variables) and the Mann-Whitney *U* or *t* test (continuous variables). Univariate risk factors that reached *p* <  0.05 were subjected to Cox regression analysis.

The nomogram was built in terms of results of Cox regression analyses using R version 3.0.2 with the rms package [19]. According to the Akaike information criterion, backward step-down selection process was applied to the selection of final model [20]. The discrimination of the models were assessed in terms of the concordance index (C-index). Bootstraps with 1000 resamples were drawn to correct the C-index. Comparisons between nomogram and other models were carried out by using the rcorrp.cens in the Hmisc package [21]. Calibration curves were also drawn to evaluate the concordance between predicted and observed probabilities. Decision curve analyses (DCA) were used to compare the benefits and improved performance of different models [22]. All tests were two-sided and were considered statistically significant when *p* <  0.05.

## Results

### Patient characteristics and outcomes

In total, 509 and 620 patients from the derivation and external validation cohorts, respectively, were included for analyses. The baseline data of enrolled patients were summarized in Table 1. In both sets, the average age was 51–52 years, and the patients were predominantly men. 410 patients (80.6%) were treated with nucleotide analogs (NUCs) and 99 patients (19.4%) didn’t received NUCs after hospital admission in the derivation cohort. 372 patients (60.0%) were treated with NUCs and 248 patients (40.0%) didn’t receive antiviral therapy in the validation cohort. The most common complication was ascites, followed by HE (I-IV) and gastrointestinal hemorrhage at admission. Liver failure was the most frequent organ failure, which was followed by coagulation and cerebral failure.

**Table 1:** Baseline characteristics in enrolled acute decompensation of CHB cirrhosis patients in the derivation and external validation cohort

Characteristic	Derivation cohort *N* = 509	External validation cohort *N* = 620	*P* value
Age (yr)	51.9 ± 11.8	51.4 ± 11.3	0.540
Male sex	359 (70.5)	463 (74.7)	0.119
Mean arterial pressure (mm Hg)	87.3 ± 10.7	87.3 ± 12.2	0.997
Previous decompensation	197 (38.7)	328 (52.9)	< 0.001
Complication			
Hyponatremia	144 (28.3)	249 (40.2)	< 0.001
Ascites	469 (92.1)	496 (80.0)	< 0.001
Bacteria infection	106 (20.8)	75 (12.1)	< 0.001
Gastrointestinal hemorrhage	123 (24.2)	62 (10.0)	< 0.001
Hepatic encephalopathy I-II	100 (19.6)	46 (7.4)	< 0.001
Hepatic encephalopathy III-IV	89 (17.5)	33 (5.3)	< 0.001
Spontaneous bacterial peritonitis	21 (4.1)	19 (3.1)	0.337
Hepatorenal syndrome	44 (8.6)	61 (9.8)	0.492
Organ failures			
Liver	96 (18.9)	152 (24.5)	< 0.001
Kidney	23 (4.5)	64 (10.3)	< 0.001
Cerebral	89 (17.5)	33 (5.3)	< 0.001
Coagulation	56 (11.0)	120 (19.4)	< 0.001
Respiratory	4 (0.8)	19 (3.1)	0.010
Circulation	6 (1.2)	9 (1.5)	0.797
Biochemical parameters			
Alanine aminotransferase (U/L)	130.7 ± 388.4	161.0 ± 428.1	< 0.001
Aspartate aminotransferase (U/L)	125.4 ± 211.3	143.0 ± 322.6	0.005
γ-Glutamyltransferase (U/L)	60.1 ± 80.9	66.5 ± 86.9	0.015
Albumin (g/L)	29.5 ± 16.3	29.7 ± 5.8	0.764
Total bilirubin (mg/dL)	6.8 ± 9.3	9.6 ± 13.7	0.004
Serum creatinine (μmol/L)	80.3 ± 58.1	89.9 ± 84.8	0.016
Serum sodium (mmol/L)	137.0 ± 5.4	134.6 ± 6.8	< 0.001
International normalized ratio	1.7 ± 1.5	2.0 ± 1.3	0.001
White blood cell count (× 10^9^/L)	5.5 ± 4.0	6.4 ± 4.8	0.009
Neutrophil count (×10^9^/L)	4.1 ± 5.8	–	–
Platelet (×10^9^/L)	73.2 ± 55.2	79.1 ± 59.2	0.240
HBV-DNA (log copies/ml)	4.1 ± 1.7	4.4 ± 1.6	< 0.001
Mortality			
7 days	19(3.7)	71(11.5)	< 0.001
14 days	40(7.9)	116(18.7)	< 0.001
28 days	56(11.0)	170(27.4)	< 0.001

Data are the mean ± standard deviation for continuous valiables, and n (%), frequency with percentage for categorical variables

When comparing the demographic and clinical characteristics between the derivation and validation sets, we found that patients in the derivation set had lower rates of previous decompensation and hyponatremia, and higher rates of complications (ascites, bacterial infection, gastrointestinal hemorrhage, HE) at the time of admission (*p* <  0.05). Moreover, the validation set had lower Na levels and higher ALT, AST, GGT, TBil, Cr, INR, WBC, and HBV DNA levels (*p* <  0.05). The validation set had higher 7-, 14- and 28-day risk of death than those of the derivation set (*p* <  0.05).

### Univariate and multivariate analyses

Univariate analysis showed that age, male sex, previous decompensation, hyponatremia, ascites, bacterial infection, gastrointestinal hemorrhage, HE III-IV, SBP, hepatorenal syndrome, organ failure (liver, kidney, cerebral, coagulation, circulatory, and lung), AST, ALB, TBil, Cr, Na, INR, WBC, and NC were significantly associated with poor prognosis of AD of CHB cirrhosis in the derivation set (*p* <  0.05, Table 2).

**Table 2:** Univariate and multivariate Cox regression analyses in patients with acute decompensation of CHB cirrhosis from the derivation cohort (n = 509)

	Univariate analysis		Multivariate analysis	
	HR (95% CI)	*p* value	HR (95% CI)	*p* value
Age (yr)	1.054(1.031–1.079)	< 0.001	1.057(1.032–1.082)	< 0.001
Male sex	2.350(1.391–3.969)	< 0.001		
Mean arterial pressure (mm Hg)	0.979(0.953–1.005)	0.116		
Previous decompensation	2.419(1.420–4.120)	0.001	2.449(1.351–4.438)	0.002
Complication				
Hyponatremia	4.005(2.351–6.823)	< 0.001		
Ascites	0.463(0.113–1.900)	< 0.001		
Bacteria infection	2.479(1.443–4.259)	0.001	5.325(3.015–9.405)	< 0.001
Gastrointestinal hemorrhage	2.301(1.042–5.081)	0.039		
Hepatic encephalopathy I-II	1.426(0.439–4.631)	0.555		
Hepatic encephalopathy III-IV	22.381(10.893–45.983)	< 0.001	4.660(3.115–6.972)	< 0.001
Spontaneous bacterial peritonitis	4.392(1.988–9.703)	< 0.001		
Hepatorenal syndrome	6.345(3.617–11.129)	< 0.001		
Organ failures				
Liver	2.720(1.583–4.672)	< 0.001		
Kidney	4.839(2.370–9.881)	< 0.001		
Cerebral	20.324(10.909–37.863)	< 0.001		
Coagulation	9.389(5.547–16.891)	< 0.001		
Circulation	10.148(3.657–28.166)	< 0.001		
Lung	9.706(3.024–31.154)	< 0.001		
Biochemical parameters				
Alanine aminotransferase (U/L)	1.000(1.000–1.001)	0.298		
Aspartate aminotransferase (U/L)	1.001(1.000–1.002)	0.005		
γ-Glutamyltransferase (U/L)	0.999(0.996–1.003)	0.722		
Albumin (g/L)	0.937(0.890–0.987)	0.015		
Total bilirubin (mg/dL)	1.048(1.029–1.067)	< 0.001	1.053(1.030–1.078)	< 0.001
Serum creatinine (μmol/L)	1.005(1.003–1.007)	< 0.001		
Serum sodium (mmol/L)	0.904(0.872–0.937)	< 0.001		
International normalized ratio	1.963(1.683–2.290)	< 0.001		
White blood cell count (×10^9^/L)	1.151(1.108–1.195)	< 0.001		
Neutrophil count (×10^9^/L)	1.175(1.128–1.225)	< 0.001		
Platelet (× 10^9^/L)	0.998(0.993–1.004)	0.576		
HBV-DNA (log copies/ml)	1.160(0.986–1.366)	0.074		

These variables were subjected to the Cox regression analyses. The results showed that only age (HR = 1.057, 95% confidence interval [CI]: 1.032–1.082, *p* <  0.001), previous decompensation (HR = 2.449, 95% CI: 1.351–4.438, *p* = 0.002), bacterial infection (HR = 5.325, 95% CI: 3.015–9.405, *p* <  0.001), HE III–IV (HR = 4.660, 95% CI: 3.115–6.972, *p* <  0.001), and TBil (HR = 1.053, 95% CI: 1.030–1.078, *p* <  0.001) were independent risk factors for outcomes (Table 2).

### Derivation of the prognostic nomogram

**Fig. 1:**
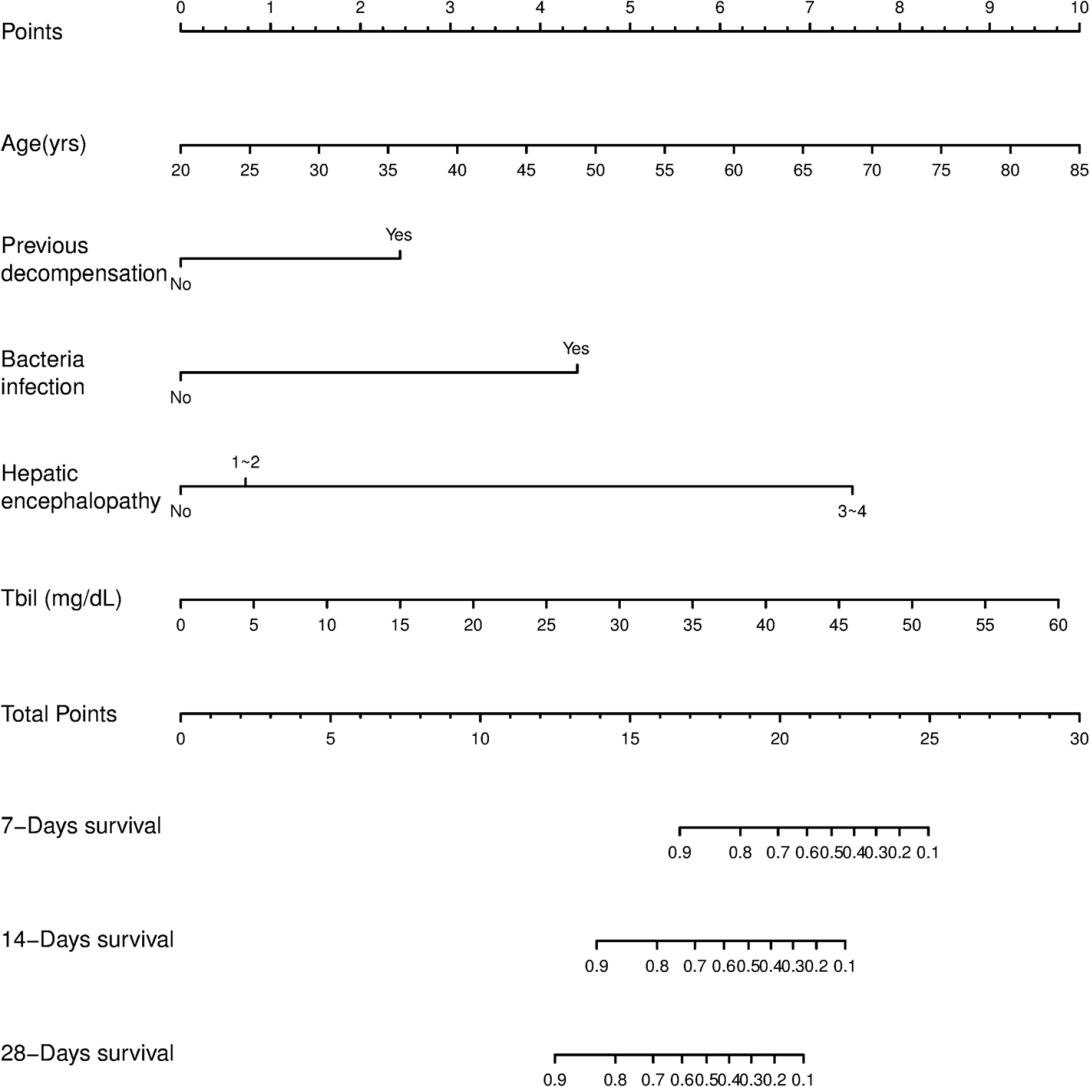
Acute decompensation of CHB cirrhosis survival nomogram. To use the nomogram, the value of an individual patient is located on each variable axis, and a line is drawn upward to determine the number of points received for the value of each variable. The sum of these numbers is located on the total point axis, and a line is drawn downward to the survival axes to determine the likelihood of 7-, 14-, and 28-day survivals

The nomogram was established on the basis of the coefficients gained from multivariate analysis, which included age, previous decompensation, bacterial infection, HE, and TBil (Fig. 1). Each value of the factors was allocated the score in the point scale axis. By summing each single score and using that value in the total point scale axis, the total score could be easily calculated to assign the probability of survival for individual patients.

### Validation of the prognostic nomogram

The C-index of the nomogram was 0.897 (95% CI: 0.850–0.943) in the derivation cohort. To more effectively validate the practicability of the nomogram, we adopted an external cohort with AD of CHB cirrhosis for model validation. When the validation set was estimated by the established nomogram, the C-index was 0.839 (95% CI: 0.811–0.867), suggesting that the nomogram is suitable for estimating the short-term outcome for AD of CHB cirrhosis.

**Fig. 2:**
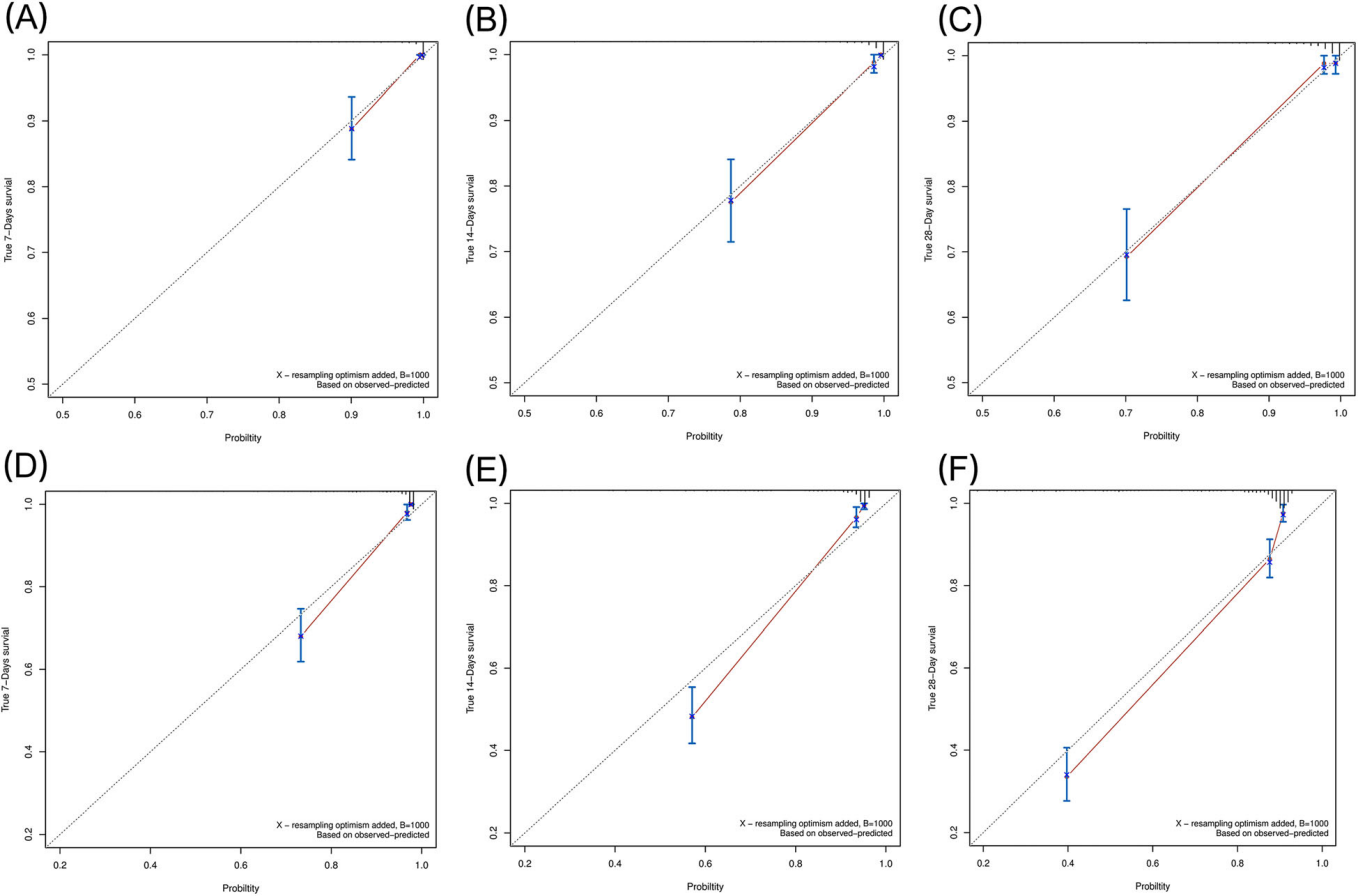
The calibration curve of overall survival at 7, 14, and 28 days for the derivation cohort (**a**-**c**) and the external validation cohort (**d**-**f**). Nomogram-predicted probability of survival is plotted on the x-axis, and the actual survival is plotted on the y-axis. Dashed lines along the 45-degree line through the point of origin represent the perfect calibration models in which the predicted probabilities are identical to the actual probabilities

The calibration curves were plotted showing that good agreements between the nomogram predictions and observed probabilities for the 7-, 14-, and 28-day outcomes in the primary (Figs. 2A-C) and external validation cohort (Figs. 2D-F).

### Performance of the nomogram compared with other models

Futhermore, we compared the C-indexes for evaluating the concordance of these models. The corrected C-index of our nomogram was 0.897, which was significantly higher than that of the MELD (0.820), CTP (0.793), SOFA (0.868), and CLIF-C ADs (0.716) in the primary set (*p* <  0.001, Table 3). Meanwhile, the nomogram also had the highest C-index (0.839) in the validation set, with statistical significance in comparison with MELD (0.826), CTP (0.741), SOFA (0.837), and CLIF-C ADs (0.569) (*p* <  0.001 for all).

**Table 3:** The predictive discrimination ability of the nomogram compared to the MELD, CTP, and SOFA score systems in the primary and validation cohorts

	C-index	95% CI for C-index		Goodness of Fit		Comparison of models			
		Lower	Upper	LR	R^2^	Dxy	SD	Z	*P* value
Primary cohort (n = 509)									
MELDs	0.820	0.764	0.878	72.07	0.185	−0.343	0.083	−4.12	< 0.001
CTPs	0.793	0.744	0.842	70.79	0.175	−0.687	0.051	−13.5	< 0.001
SOFAs	0.868	0.829	0.907	119.46	0.282	−0.398	0.075	−5.33	< 0.001
CLIF-C ADs	0.716	0.636	0.796	45.36	0.121	−0.476	0.078	−6.10	< 0.001
Nomogram	0.897	0.850	0.943	165.63	0.374	–	–	–	
Validation cohort (n = 620)									
MELDs	0.826	0.794	0.857	238.69	0.336	0.451	0.045	10.13	< 0.001
CTPs	0.741	0.707	0.776	130.11	0.196	−0.557	0.035	−15.94	< 0.001
SOFAs	0.837	0.807	0.866	255.24	0.349	−0.215	0.049	−4.39	< 0.001
CLIF-C ADs	0.569	0.525	0.615	8.47	0.015	−0.501	0.041	−12.22	< 0.001
Nomogram	0.839	0.811	0.867	268.38	0.363	–	–	–	

**Fig. 3:**
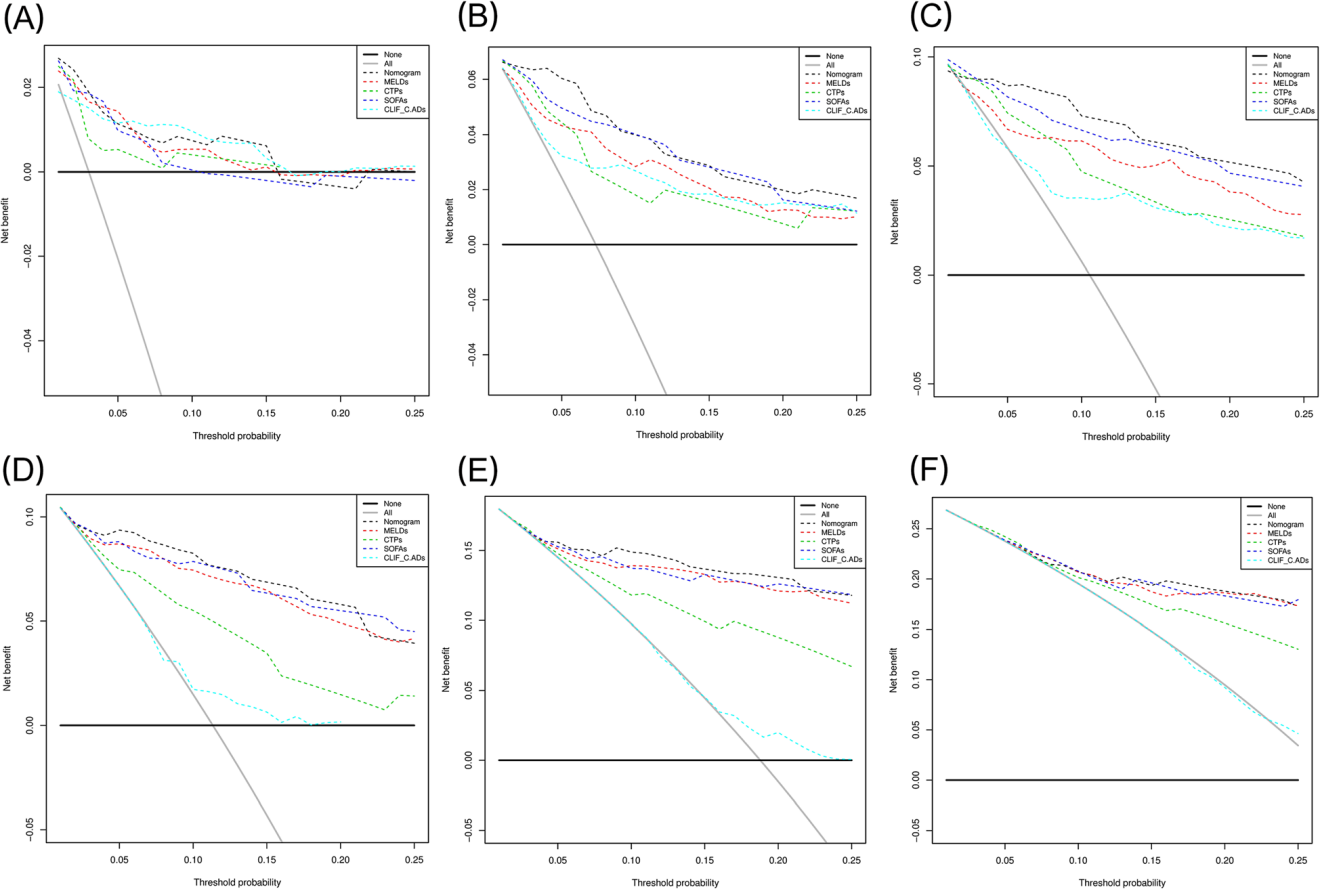
Decision curve analysis at 7, 14, and 28 days for the derivation cohort (**a**-**c**) and the external validation cohort (**d**-**f**). Decision curve analyses depict the clinical net benefit in pairwise comparisons across the different models. The horizontal solid black line represents the assumption that no patients will experience the event, and the solid gray line represents the assumption that all patients will relapse. On decision curve analysis, the nomogram showed superior net benefit compared with other models across a range of threshold probabilities

On DCA, our nomogram provided superior net benefit and improved performance for the 7-, 14-, and 28-day prognostic evaluation in the primary (Figs. 3A-C) and validation (Figs. 3D-F) cohorts relative to MELD, CTP, SOFA, and CLIF-C AD score models. This represents superior clinical usefulness of the nomogram over other score models.

## Discussion

In the present study, a novel and easy-to-use nomogram was constructed to evaluate individual prognosis for AD of CHB cirrhosis. This nomogram demonstrated superior predictive capability and clinical usefulness relative to the current prognostic score models, including MELD, CTP, SOFA, and CLIF-C ADs.

In our derivation cohort, 11.0% (56/509) of patients with AD of CHB cirrhosis died within 4 weeks—a result that was similar to that of another study [23]. A total of 13.0% (66/509) of the patients died within 90 days, and 56 of 66 patients died in the first 4 weeks; thus, we set up the 28-day statistical prognostic score model for patients with AD to find out those at the highest risk for death, which would enable us to take effective treatment measures as soon as possible. In addition, most of studies on prognosis factors for liver decompensation cirrhosis did not strictly distinguish the etiology, such as viral hepatitis, alcohol, drugs, or other factors [17, 24]. Our study only observed patients with AD of CHB cirrhosis as subjects; both blood biochemical tests and clinical complications were part of our prognosis factors. We also excluded the influence of liver transplantation, considering this has less application in our country. Moreover, to effectively evaluate the predictive ability of our nomogram versus those of established models, we adopted the external validation cohort to reduce the influence of drugs, treatments, and other factors on the results.

The proposed nomogram included age, two liver function indices (previous decompensation, TBil), and two complications (bacterial infection and HE). Many studies have suggested the correlation between age and prognosis in many diseases, and that old age is an important factor in poor prognosis for cirrhosis [25, 26]. Recently, the CANONIC study team built two models for ACLF (CLIF-C ACLFs) and AD (CLIF-C ADs) patients, where old age was considered an important indicator of poor outcomes [5, 15]. This may be explained by loss of immune function or decline in tissue regeneration and repair [27, 28]. Serum TBil is commonly used as an indicator of the degree of liver damage and reserve function; it is also the main parameter of the CTP and MELD score systems [16, 17]. Previous decompensation could better reflect the underlying disease before the onset of AD. Bacterial infection represent the particularly important cause of liver failure and other complications [29]. The end-organ damaging effect of bacterial infection is more serious in liver cirrhosis and often culminates in newly developed liver and extrahepatic organ failures, which account for significant morbidity and mortality [4, 30]. HE is a frequent and serious complication of cirrhosis [31, 32]. Once HE occurs in patients with cirrhosis, the prognosis is poor, and liver transplant should be performed as soon as possible in order to increase the survival rate [33, 34].

In this study, the nomogram model performed well in predicting survival, as supported by the C-index (0.897 and 0.839 for the primary and validation cohorts respectively) and the calibration curve. Compared with MELD, CTP, SOFA, and CLIF-C ADs, the nomogram showed superior predictive capability for outcomes. Furthermore, the nomogram demonstrated better net benefit and improved performance for 7-, 14-, and 28-day prognostic evaluation in the derivation and validation cohorts compared with MELD, CTP, SOFA, and CLIF-C AD score models.

In current study, some patients didn’t received antiviral therapy. One of the reasons is that patients with HE III-IV and gastrointestinal hemorrhage are unable to take drugs. Another reason is that the antiviral therapy was administered not only according to HBV replication levels, but also the willingness of the patient. So, considering the bad financial condition, many patients have to abandon the use of antiviral drugs. Besides, more patients didn’t received the antiviral therapy in the validation cohort. The major reason is that the enrollment time of patients in validation cohort is from January 2005 to December 2010, four years ahead of the derivation cohort. Antiviral drugs are more widespread with the development of healthcare in China. This reflects the clinical treatment situation of China objectively.

Our study has several limitations. First, our nomogram only included basic laboratory data. However, this study aimed to construct a reliable prognostic model. To avoid inevitable bias, subjective variables have not been included to construct our nomogram. Second, the nomogram was built on the basis of a retrospective cohort, and selection bias may exist. However, we have validated the model with data from another institution. The results consistently demonstrated the very good performance of our established nomogram.

## Conclusions

To our knowledge, the nomogram model here is the first model developed to predict the individual prognosis of AD of cirrhosis in CHB patients to date. This provided better performance than MELD, CTP, SOFA, and CLIF-C AD scores, and it offers a foundation for individualized counseling and clinical treatment.

## Abbreviations

AD: acute decompensation
ALB: albumin
ALP: alkaline phosphatase
ALT: alanine aminotransferase
AST: aspartate aminotransferase
CHB: chronic hepatitis B
CIs: confidence intervals
CLIF-C AD: Chronic Liver Failure Consortium Acute Decompensation
CTP: Child-Turcotte-Pugh
DCA: decision curve analysis
GGT: ɣ-glutamyltranspeptidase
HBV: hepatitis B virus
HE: hepatic encephalopathy
HRs: hazard ratios
INR: international normalized ratio
MELD: Model for End-stage Liver Disease
NC: absolute neutrophil count
PLT: absolute platelet count
SOFA: Sequential Organ Failure Assessment
TBil: total bilirubin
WBC: white blood cell
